# Genome-Wide Exome Analysis of *Cmv5*-Disparate Mouse Strains that Differ in Host Resistance to Murine Cytomegalovirus Infection

**DOI:** 10.1534/g3.117.042531

**Published:** 2017-04-26

**Authors:** Alyssa Gillespie, Heather Lee, Catherine Robertson, Maya Cabot, Michael G. Brown

**Affiliations:** * Department of Medicine, Division of Nephrology, University of Virginia School of Medicine, Charlottesville, Virginia 22908; † Beirne B. Carter Center for Immunology Research, University of Virginia School of Medicine, Charlottesville, Virginia; ‡ Department of Biology, University of Virginia School of Medicine, Charlottesville, Virginia; § Department of Microbiology, Immunology, and Cancer Biology, University of Virginia School of Medicine, Charlottesville, Virginia; ** Department of Molecular Physiology and Biological Physics, University of Virginia School of Medicine, Charlottesville, Virginia 22908

**Keywords:** MA/My, C57L, M.H2^b^, MHC, QTL, NK cell, Ly49, Genetics of Immunity

## Abstract

Host resistance to murine cytomegalovirus (MCMV) varies in different strains of laboratory mice due to differences in expression of determinants that control and clear viral infection. The major histocompatibility complex class I D^k^ molecule is one such determinant that controls MCMV through the action of natural killer (NK) cells. However, the extent of NK cell–mediated D^k^-dependent resistance to infection varies in different mouse strains. The molecular genetic basis of this variation remains unclear. Previous work to examine the D^k^ effect on MCMV resistance in MA/My × C57L offspring discovered multiple quantitative trait loci (QTL) that may serve to modify NK cells or their capacity to respond during MCMV infection. One QTL in particular, *Cmv5*, was found to regulate the frequency of NK cells and secondary lymphoid organ structure in spleen during MCMV infection. *Cmv5* alleles, however, have not been identified. We therefore sequenced and analyzed genome-wide exome (GWE) variants, including those aligned to the critical genetic interval, in *Cmv5*-disparate mouse strains. Their GWE variant profiles were compared to assess strain-specific sequence data integrity and to analyze mouse strain relatedness across the genome. GWE content was further compared against data from the Mouse Genomes Project. This approach was developed as a platform for using GWE variants to define genomic regions of divergence and similarity in different mouse strains while also validating the overall quality of GWE sequence data. Moreover, the analysis provides a framework for the selection of novel QTL candidate sequences, including at the *Cmv5* critical region.

The major histocompatibility complex (MHC) class I D^k^ molecule is a potent resistance factor that protects MA/My mice during infection with murine cytomegalovirus (MCMV) (Dighe *et al.* 2005; Desrosiers *et al.* 2005; Xie *et al.* 2010; Fodil-Cornu *et al.* 2011). Expression of a genomic D^k^ transgene in C57L.D^k^ (L.D^k^) mice similarly protects otherwise susceptible C57L mice during MCMV infection (Xie *et al.* 2010). Interestingly, D^k^ is a specific ligand of the Ly49G2 inhibitory receptor, and D^k^-dependent resistance in MA/My and L.D^k^ mice also requires Ly49G2-positive natural killer (NK) cells (Xie *et al.* 2009, 2010). Ly49G2-positive NK cells that are exposed to D^k^ during development acquire increased responsiveness and the capacity to mediate MCMV resistance (Wei *et al.* 2014; Nash *et al.* 2014) through an educational process referred to as NK cell licensing (Fernandez *et al.* 2005; Kim *et al.* 2005). However, this D^k^-dependent resistance effect mediated by Ly49G2-positive NK cells varies by genetic background. While MA/My.L-H2^b/k^ (M.H2^het^) mice are more resistant to MCMV than their MA/My.L-H2^b^ (M.H2^b^) congenic counterparts, they are also more susceptible to infection than either MA/My or C57L.M-H2^b/k^ (L.H2^het^) mice (Xie *et al.* 2007). These data suggest that NK cell–mediated resistance to MCMV can be modified in different strains with D^k^ expressed, but the molecular genetic basis of variation in resistance is not understood.

We used a deep phenomic approach to assess the effects of genomic modifiers or quantitative trait loci (QTL) on immune cell and response features before and after infection in MA/My × C57L mice (Gillespie *et al.* 2016; Prince *et al.* 2013). We discovered that *H-2D* has a profound and specific effect on the frequency of Ly49G2-positive NK cells and inhibitory receptor expression on individual NK cells in naive mice (Gillespie *et al.* 2016). Moreover, *H-2D* was found to control the Ly49G2-positive NK cell response, weight change, and virus burden in spleen and liver tissues during MCMV infection. D^k^-dependent control of Ly49G2-positive NK cell frequency and responsiveness therefore results in less morbidity and greater overall host resistance during MCMV infection. However, both the overall frequency of NK cells and secondary lymphoid organ structures in the spleen during MCMV infection are affected by a different QTL, namely *Cmv5*, which maps distal to *H-2D* (Gillespie *et al.* 2016). An intriguing aspect of the C57L-derived *Cmv5* susceptibility allele is that it suppresses D^k^-dependent MCMV resistance. However, causative genetic variants have yet to be ascribed to *Cmv5* or any other previously mapped QTL.

As one approach toward discovering genetic variants of interest, we examined genome-wide exome (GWE) sequences in *Cmv5*-disparate strains MA/My, C57L, and M.H2^b^. GWE sequence content was aligned to C57BL/6 (B6) and then further compared to the relevant strains. We assessed GWE sequence quality, reliability, and overall extent of variability among the strains. *Cmv5*-disparate GWE profiles were further compared against a panel of common laboratory strains in the Mouse Genomes Project (MGP) to glean additional insight about the quality of GWE sequences, strain-dependent relatedness, and putative *Cmv5* candidates.

## Materials and Methods

### Mice

MA/My and C57L mice were purchased from The Jackson Laboratory and maintained at the University of Virginia (UVA). M.H2^b^ congenic mice were generated previously (Xie *et al.* 2007) and have been bred and maintained at UVA. Experiments conducted in this study were performed in accordance with the Animal Welfare Act and the recommendations in the Guide for Care and Use of Laboratory Animals of the National Institutes of Health, and were approved by the UVA Animal Care and Use Committee (protocol #3050).

### DNA preparation and exome sequencing

To identify high probability gene candidates for previously mapped QTL, genome-wide sequencing of mouse exomes was performed. DNA was extracted from MA/My (liver), C57L (liver), and M.H2^b^ (spleen and lung) female tissues (14–22 wk old) using a kit (Gentra Systems). GWE sequencing was performed at Hudson-Alpha Institute for Biotechnology (Huntsville, Alabama). Briefly, 2 μg of intact genomic DNA was fragmented by sonic shearing using a Covaris LE220. DNA fragments were hybridized to the NimbleGen SeqCap EZ Target Enrichment System Exome Library. Complexes of library oligos and DNA fragments were captured, purified, and then enriched by PCR. Samples were run on the Illumina HiSequation 2000. The DRAGEN Genome sequencing analysis pipeline was used to perform sequence alignment and variant calling based on the B6 GRCm38/mm10 reference genome (http://www.edicogenome.com/pipelines/dragen-genome-pipeline/). Average depth of coverage across MA/My and C57L exomes was ∼10 times, and the M.H2^b^ exome was ∼17 times. Binary alignment map (BAM) files with raw sequence data have been uploaded to the NCBI Sequence Read Archive (accession no.: SRP077658). BAM file comparisons to the B6 reference genome yielded exome variant call files (VCFs) for each strain, also provided by Hudson Alpha.

GWE variants, representing sites where a given strain differs from the B6 reference strain, were filtered for quality according to several criteria (Supplemental Material, File S1). After filtering, 496407, 228732, and 387357 variants were detected in the MA/My, C57L, and M.H2^b^ strains, respectively. C57L had the fewest variants, which is indicative of its close genealogical relatedness to B6 (Beck *et al.* 2000).

### GWE sequence analysis workflow

VCFs were analyzed using Ensembl Variant Effect Predictor (VEP) to assess variation in MA/My, C57L, and M.H2^b^ exomes. VEP files (File S2) were loaded as data frames into R using [read.csv]. A source file containing R code is also provided (File S3). Low and modifier impact variants, duplicate variants, and mitochondria DNA variants were removed using [subset]. Chromosome “X” and deletions “-” were renamed “20” and “D,” respectively, and bases were converted to Mb. VEP data frame comparisons were performed in R, using [sqldf] to identify common and strain-specific exome variants. Variant numbers per 10-Mb bin were enumerated for all bins on each chromosome [data.frame(table(cut(breaks = seq(0, 200, by = 10))))]. Data frames of enumerated bins were merged using [list], [reduce], and [merge], resulting in a master data frame with both total and strain-specific variant numbers per bin. The sum of specific variants for combinations of two strains (*e.g.*, MA/My-specific variants plus C57L-specific variants) was calculated using [transform]. Heatmaps depicting total variant numbers per strain and the sum of strain-specific variants per two strain combinations were created in R using [ggplot2] and [gridExtra]. Histograms of the frequency distribution of total variants per 10-Mb bin for each strain were generated by using [melt] in the [reshape package] on total variant numbers per bin to create a data frame with columns for strain and number of variants per bin. Rows with 0 variants/bin were removed using [subset]. Log_2_(variants/ bin) calculated in R using [transform] and a data frame containing median value for each GWE distribution was generated using [aggregate]. Figures depicting the results were created in R using [ggplot2].

### Comparative analysis of MHC-linked exome sequences

Original data frames were reduced to the *Cmv5* interval (*Rrp1-Sgol1*) using [subset(Chr == 17 & Pos >32.05 & Pos <53.68)]. Allele columns in each data frame were renamed to the strain names using [paste]. Additional data frames were created in R using [sqldf], including variants (*i.e.*, in relation to B6) common to MA/My and C57L for C57L; variants common to MA/My and C57L for MA/My; variants common to MA/My, C57L, and M.H2^b^; MA/My-specific variants for MA/My; MA/My-specific variants for M.H2^b^; C57L-specific variants for C57L; C57L-specific variants for M.H2^b^; and M.H2^b^-specific variants. All eight data frames were necessary for figure creation. Nucleotide calls in each data frame were assigned a variant number based on type: those common to MA/My and C57L, and those that are MA/My-, C57L-, or M.H2^b^-specific. Data frames were then merged in R, which resulted in three data frames containing variants common to MA/My and C57L in addition to those that are MA/My-, C57L-, or M.H2^b^-specific and their variant type number. These were further merged into a single data frame and converted using [melt] to contain columns for chromosome, position, gene, strain, and variant type number. A figure depicting MHC-linked exome (MLE) variants in the *Cmv5*-disparate strains was also created in R using [ggplot2] and color-coded by variant type.

For MLE sequence comparisons to genomic content of MGP strains, variants aligned to the *Cmv5* interval for all MGP strains were acquired and loaded into R as a data frame. Duplicates were removed, and bases were converted to Mb. MA/My, L-UVA, and M.H2^b^ data frames were reduced to the same interval using [subset(Chr == 17 & Pos >32.05 & Pos <53.68)]. Columns were reordered to reflect MGP data frame organization with unnecessary columns removed. Allele columns for each data frame were renamed strain names using [paste]. The data frames were then merged into one. All “NA” text descriptors were converted to “0,” and all variants were converted to “1.” The data frame was then reduced to MA/My-specific variants (*i.e.*, in relation to B6) using [subset]. MA/My-defining MLE variants were compared to all other strains in R and new data frames were created to report on each strain and the frequency of MLE variants that were in common with MA/My. A bar graph depicting the percentage match with MA/My was created in R using [ggplot2].

### Data availability

Mouse strains are available upon request. BAM files with raw sequence data and VCFs are available at the NCBI Sequence Read Archive (accession no.: SRP077658). File S1 provides a workflow for GWE sequence variant filtering. File S2 provides a zip folder of VEP files for the sequenced strains. File S3 provides the R code used to analyze VEP files.

## Results

### Sequencing and genome-wide analysis of exome variants

**Figure 1:**
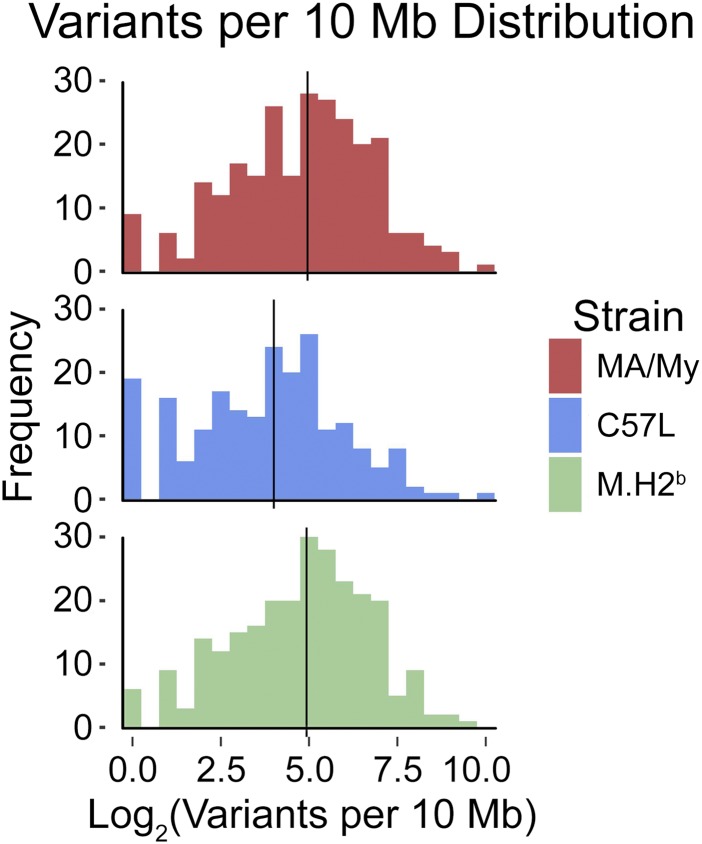
Mouse strain-specific distribution of genome-wide exome variants in relation to B6. Exome variants in relation to B6 were enumerated for 10-Mb bins on every chromosome. Shown for each strain is a frequency distribution plot of exome variants per bin and its median value (black line), across the genome.

We focused our analysis on genes with high or moderate impact variants that are more likely to result in significant functional changes. As a result, strain-specific GWE variants were compiled for each of the relevant strains. The extent of GWE variation per strain was analyzed by enumerating variants per 10-Mb bins across every chromosome. Similar GWE distribution profiles, in relation to B6, highlight a high degree of similarity obtained for MA/My and M.H2^b^ strains (Figure 1). A shift to the right in the MA/My-related GWE distributions with higher median values in relation to the C57L profile suggests that analysis of genome diversity may reveal ancestral relationships and relevant genetic candidates. We infer that most bins in each strain had sufficiently diverse pools of exome sequences, and that C57L retains more B6-like GWE content than either of the MA/My-derived strains.

**Figure 2:**
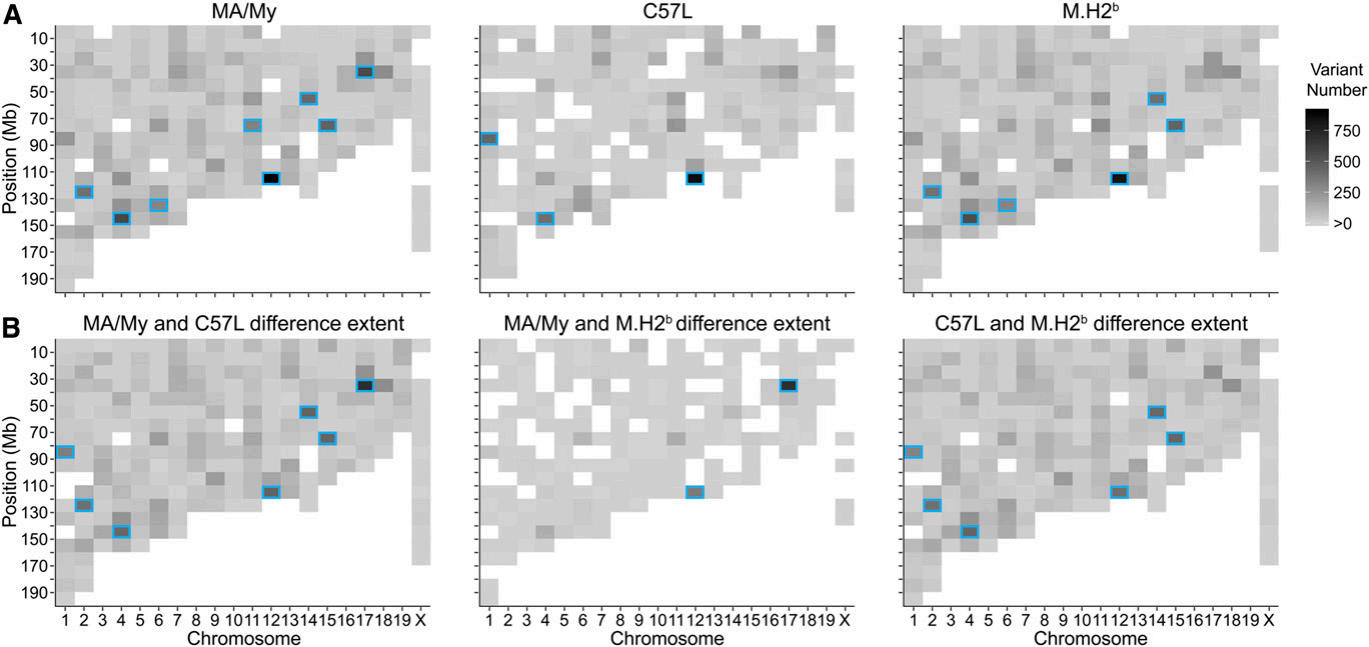
Comparative analysis of GWE variability among *Cmv5*-disparate mouse strains. (A) The heatmaps show strain-specific profiles of the number of exome variants per 10-Mb bin across every chromosome in relation to B6. (B) As in (A), but the heatmaps display total strain-specific GWE variants per 10-Mb bin for the indicated strains. High diversity (300+ exome variants) chromosome bins (blue boxes) that distinguish B6, MA/My, and/or C57L GWE profiles are marked.

As a more rigorous assessment of sequence quality and the extent of GWE variation, strain-specific exome variant numbers relative to B6 were plotted by chromosome. Variation in MA/My exomes were observed broadly across the genome, with most chromosome bins having <50 variants (Figure 2A). Greater diversity in some bins, however, readily distinguished the GWE profile in MA/My from B6. Expected high variation in the MHC region (*i.e.*, bin 30–40 Mb on chr-17) distinguished MA/My from B6 (Figure 2A). The same region in C57L and M.H2^b^ strains was found to be much more similar to B6, consistent with the introgression of a C57L-derived chr-17 fragment in M.H2^b^ (Xie *et al.* 2007). Nonetheless, beyond the MHC difference, GWE profiles in MA/My and M.H2^b^ proved to be highly related (Figure 2A). Direct comparison of MA/My and M.H2^b^ GWE profiles verified this point because many bins lacked any difference, and only the MHC on chr-17 and another position on chr-12 had substantial differences (Figure 2B). The latter bin on chr-12 corresponding to the IgH chain locus distinguished all of the strains, consistent with significant exome sequence diversification. MA/My and M.H2^b^ diversity in this interval may be related to the use of M.H2^b^ spleen DNA. Several additional high diversity bins on chr-1 (*Fam124b-Dock10-Itm2c-Chrng-Ngef*), chr-2 (β*2m-Sirp*α), chr-4 (*Tnfrsf* multigene locus), chr-6 (NK gene complex), chr-11 (*Itgae-Hic1*), chr-14 (*Tcr* α*/*δ locus), and chr-15 (*Ly6* multigene locus) also distinguished MA/My or C57L from B6 and/or each other (Figure 2, A and B). In contrast, GWE profiles in MA/My and M.H2^b^ both differed from C57L in similar ways across the genome. In aggregate, these data suggest that GWE sequencing produced high quality data, which was useful for assessing genomic diversity and mouse strain relatedness.

### Comparative analysis of MLE profiles corresponding to the Cmv5 critical region

The Sanger Center recently released the MGP with short-read sequence variants (SNPs, short insertions and deletions, and larger structural variations) for 36 different mouse strains, including C57L, with an average depth of coverage of the genome of 64 times (Keane *et al.* 2011). Because MGP sequence alignment and variant calling used B6 as its reference genome, we compared GWE variants for C57L mice bred in-house at UVA (L-UVA) to C57L/J obtained by the MGP (L-MGP) to glean additional insight into the quality of the data sets. We observed that 93% of L-UVA and L-MGP SNPs were shared. However, only 74% of GWE variants residing within L-MGP coding sequence regions (∼34 Mb, as defined by Gencode vM10) were also detected in L-UVA. This discrepancy may be due to inefficient enrichment of certain coding regions by exome capture. Nonetheless, as expected, concordance was much lower when comparing MA/My or M.H2^b^ to either L-UVA or L-MGP.

**Figure 3:**
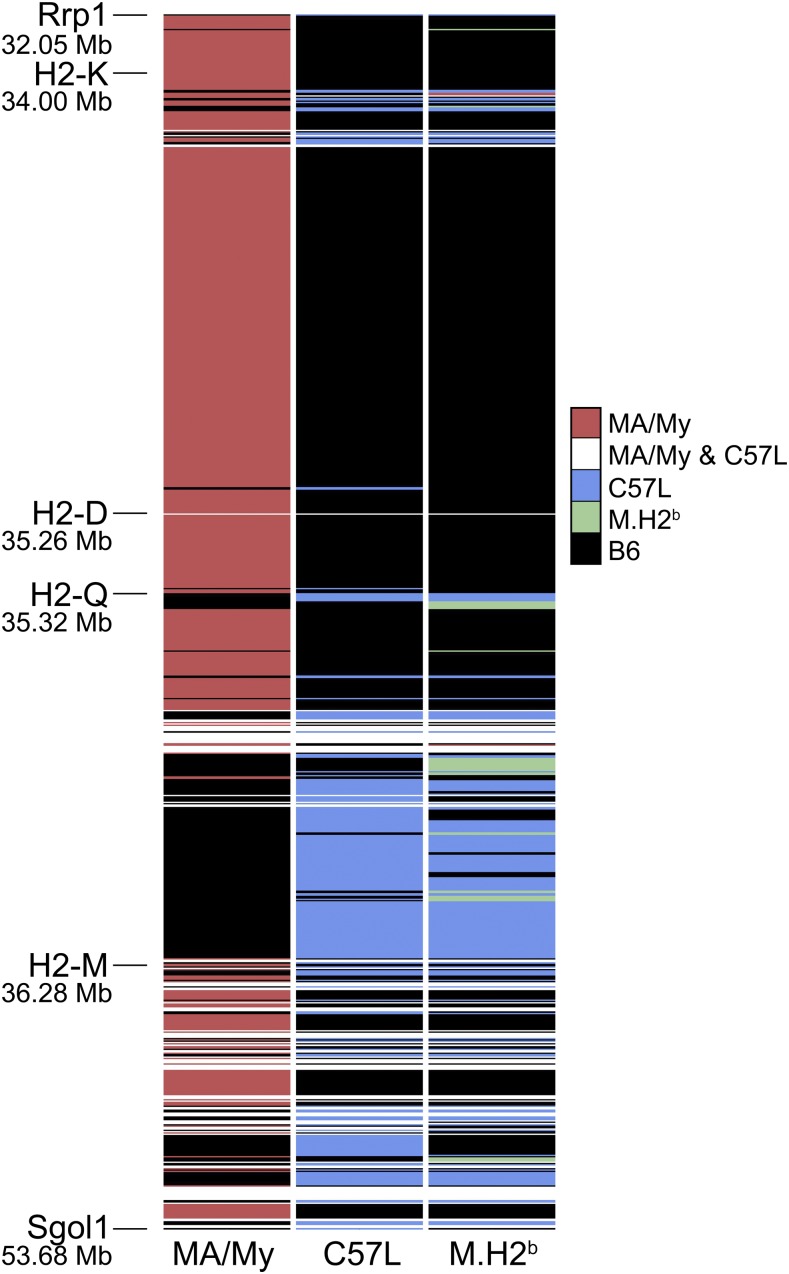
Visualization of MLE variability in *Cmv5*-disparate mouse strains. The *Cmv5* interval (*Rrp1-Sgol1*) is depicted by displaying MHC-linked exome variants detected for each of the indicated strains. MA/My- (red), C57L- (blue), and M.H2^b^-specific (green) exome variants in relation to B6 are shown. MLE sequences identical to B6 (black) and those detected only in the *Cmv5*-disparate strains (white) are also shown. Chr-17 positions (megabases, Mb) of several genes are shown on the map on the left; however, this is not a physical map since only exome variants for the indicated strains are included.

We next compared MLE profiles corresponding to the *Cmv5* (*Rrp1b-Sgol1)* interval on chr-17 in MA/My, L-UVA, and M.H2^b^. In total, MA/My had 670 MLE variants relative to B6, while L-UVA and M.H2^b^ had 319 and 306, respectively (Figure 3). Of the variants identified in L-UVA and M.H2^b^, 270 are common. The results validate substantial MLE variation in MA/My and B6, while C57L and M.H2^b^ MLE profiles are much more similar to B6, and even more related to each other (Figure 3). Only a minor fraction of all MLE variants analyzed were detected solely in L-UVA (5.3%) or M.H2^b^ (3.9%), which suggests that GWE sequence artifacts, undetected cross-over events, and/or strain-specific sequence variants may have been acquired.

**Figure 4:**
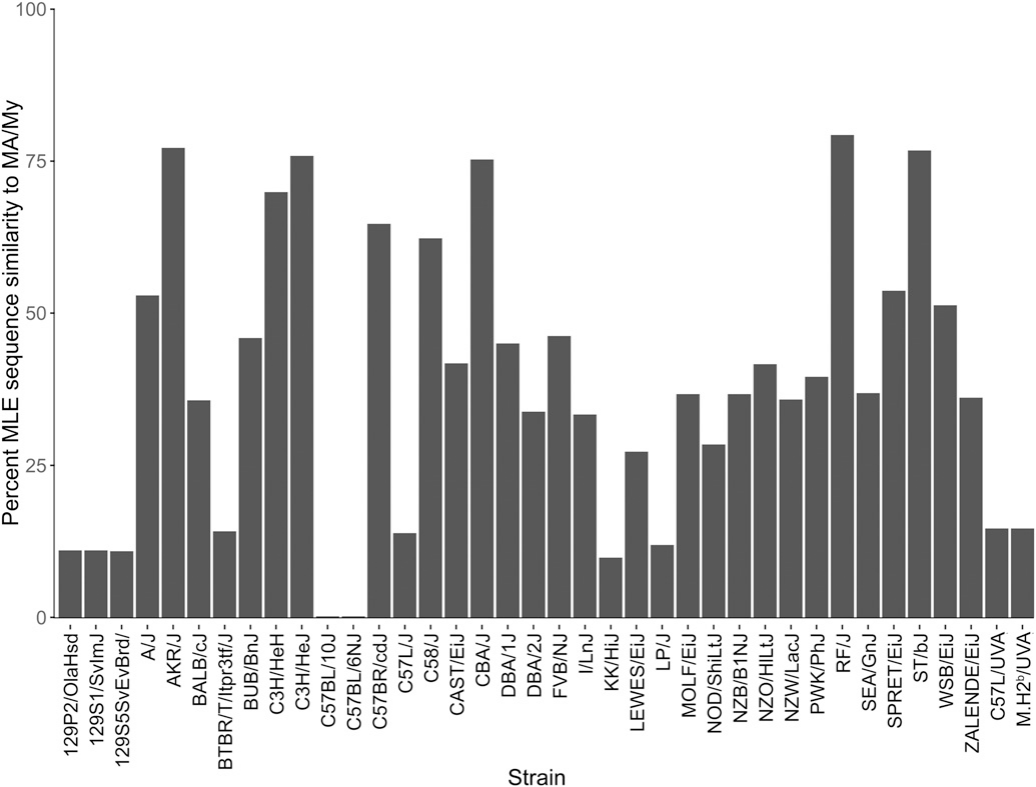
The extent of MHC-linked exome variability in MGP strains in relation to MA/My and alignment of exome variants to the *Cmv5* critical region. High and moderate impact exome variant sequences from the *Cmv5*-disparate strains that aligned at the *Cmv5* interval (*Rrp1b-Sgol1*) were compared against genomic sequences obtained for all 36 MGP strains in the statistical computing program R. The graph shows the percentage of MA/My variants (mapped relative to B6) that are common to each of the indicated strains.

Although the MGP does not include MA/My genome sequence, we further compared MA/My, L-UVA, and M.H2^b^ to MGP MLE profiles using the statistical computing program R. We found that the MHC region in the RF strain of William E. Castle’s group B mice (Beck *et al.* 2000) is more related (79.25% MLE identity) to MA/My than any other mouse strain analyzed in the MGP, although profile similarity scores for other H2^k^ strains, including AKR/J, C3H/HeJ, CBA, and ST/bJ (Fischer Lindahl 1997), also exceeded 75% similarity (Figure 4). Whereas group E C57-related strains were included in crosses with Castle’s group B strains (Beck *et al.* 2000), it appears likely that strain RF acquired its MHC haplotype from a group E strain. Although slightly greater MLE variability was detected in C3H/HeH, C57BR, and C58 strains, they too have retained MA/My-like (*i.e.*, H2^k2^) features. On the other hand, strains 129, BTBR, C57BL, C57L/J (MGP), KK, LP, C57L (L-UVA), and M.H2^b^ display much greater MLE variability in comparison to MA/My (Figure 4), consistent with known MHC haplotype disparity in the strains (Fischer Lindahl 1997).

## Discussion

Here, we analyzed the extent of GWE variability in *Cmv5*-disparate mouse strains to examine mouse strain relatedness, and to discover *Cmv5* candidate exome sequences. A primary concern centered on evaluating GWE sequence quality. Despite the fact that genomic sequence data for two of the key strains is not publicly available, we implemented several strategies to assess GWE sequence data in preparation for selection of *Cmv5* candidates.

Because the M.H2^b^ congenic strain had been constructed by crossing MA/My and C57L mice, we examined GWE profiles for these strains. As expected, very low GWE variability was detected at chromosome regions other than chr-17 in MA/My-related strains, whereas MLE sequences were highly discordant (Figure 2 and Figure 3). In contrast, M.H2^b^ and C57L GWE profile comparisons revealed higher variability for much of the genome, except at the MHC on chr-17. Therefore, our comparative analyses provide an independent measure of GWE sequence quality, and further establish this method as a strategy to discover GWE sequence variants as positional candidates for mapped QTL. However, this gene-discovery approach suffers from many challenges ascribed to positional cloning strategies used to identify QTL (Andrews *et al.* 2012; Flint *et al.* 2005), including those based on analysis of extant mouse strains using classical genetics (Poltorak *et al.* 1998; Brown *et al.* 2001; Xie *et al.* 2010), site-directed mutagenesis of the well-known B6 mouse strain (Nelms and Goodnow 2001; Hoebe *et al.* 2003), and the more recent collaborative cross of eight different laboratory strains of mice (Churchill *et al.* 2004; Threadgill and Churchill 2012). Issues related to poor sequence quality, poor coverage including regulatory sequence variants missed by exome analysis, sequence misassembly, and strain-related haplotype variation can therefore complicate the process of identifying a causal *Cmv5* allele.

We further assessed *Cmv5*-disparate MLE sequences by comparison to genomic sequence content for 36 additional mouse strains examined in the MGP. We found that MLE sequences in MA/My are most similar in genomic content to H2^k^ and H2^k2^ strains. Much greater variability was observed upon comparison of MA/My MLE sequences to genomic sequences of H2^d^ (BALB/cJ, DBA/2J, SEA_GnJ) and H2^bc^ (129-related strains, and LP_J) strains. MA/My MLE sequences also differ substantially from H2^b^ strains, including BTBR_T_ltpr3tf_J, C57L/J (*i.e.*, L-MGP), C57L-UVA, and M.H2^b^; however, the extent of variation differs when compared to the H2^b^ strains C57BL/6NJ and C57BL/10J, which suggests that MHC-linked loci have further diversified in different H2^b^-related strains since strain separation. A viable approach for selection and analysis of GWE sequences as QTL candidates, and a genomic framework and context for discovery of *Cmv5* alleles is established. We envisage leveraging it with precision QTL mapping and complementary gene expression and sequencing analyses, which could lead to *Cmv5*’s discovery.

## Supplementary Material

Supplemental material is available online at www.g3journal.org/lookup/suppl/doi:10.1534/g3.117.042531/-/DC1.
