# Supplementary material for: Genome-Wide Exome Analysis of Cmv5-Disparate Mouse Strains that Differ in Host Resistance to Murine Cytomegalovirus Infection
Source: G3 (Bethesda). 2017 Apr 26;7(6):1979–84. doi: 10.1534/g3.117.042531 (PMC5473773; doi:10.1534/g3.117.042531)
Supplement: Supplementary file 1 [file 1979FileS1.docx]

**Supplemental File S1.**

**GWE sequence variant filtering criteria.** Variants meeting one or more of these criteria were excluded from further analysis of genomic diversity.

| **SNP exclusion criteria** | **Indel exclusion criteria** |
| --- | --- |
| Variance confidence/Quality by depth < 2.0  RMS mapping quality < 40.0  Phred-scaled p-value using Fisher’s exact test to detect strand bias > 60.0  Z-score from Wilcoxon rank sum test of alternative versus reference read mapping qualities < -12.5  Z-score from Wilcoxon rank sum test of alternative versus reference read position bias < -8 | Variance confidence/Quality by depth < 2.0  Phred-scaled p-value using Fisher’s exact test to detect strand bias > 200.0  Z-score from Wilcoxon rank sum test of alternative versus reference read position bias < -20.0 |
